# Computational Complexity Reduction of Neural Networks of Brain Tumor Image Segmentation by Introducing Fermi–Dirac Correction Functions

**DOI:** 10.3390/e23020223

**Published:** 2021-02-11

**Authors:** Yen-Ling Tai, Shin-Jhe Huang, Chien-Chang Chen, Henry Horng-Shing Lu

**Affiliations:** 1 Bio-Microsystems Integration Laboratory, Department of Biomedical Sciences and Engineering, National Central University, Taoyuan City 32001, Taiwan; greenni630@gmail.com (Y.-L.T.); handsome410115@yahoo.com.tw (S.-J.H.); gettgod@ncu.edu.tw (C.-C.C.); 2 Chronic Disease Research Center, National Central University, Taoyuan City 32001, Taiwan; 3 Shing-Tung Yau Center, National Chiao Tung University, Hsinchu 30010, Taiwan; 4 Institute of Statistics, National Chiao Tung University, Hsinchu 30010, Taiwan; 5 Institute of Statistics, National Yang Ming Chiao Tung University, Hsinchu 30010, Taiwan

**Keywords:** computational complexity, dimensional fusion U-net, Fermi–Dirac distribution, image segmentation

## Abstract

Nowadays, deep learning methods with high structural complexity and flexibility inevitably lean on the computational capability of the hardware. A platform with high-performance GPUs and large amounts of memory could support neural networks having large numbers of layers and kernels. However, naively pursuing high-cost hardware would probably drag the technical development of deep learning methods. In the article, we thus establish a new preprocessing method to reduce the computational complexity of the neural networks. Inspired by the band theory of solids in physics, we map the image space into a noninteraction physical system isomorphically and then treat image voxels as particle-like clusters. Then, we reconstruct the Fermi–Dirac distribution to be a correction function for the normalization of the voxel intensity and as a filter of insignificant cluster components. The filtered clusters at the circumstance can delineate the morphological heterogeneity of the image voxels. We used the BraTS 2019 datasets and the dimensional fusion U-net for the algorithmic validation, and the proposed Fermi–Dirac correction function exhibited comparable performance to other employed preprocessing methods. By comparing to the conventional z-score normalization function and the Gamma correction function, the proposed algorithm can save at least 38% of computational time cost under a low-cost hardware architecture. Even though the correction function of global histogram equalization has the lowest computational time among the employed correction functions, the proposed Fermi–Dirac correction function exhibits better capabilities of image augmentation and segmentation.

## 1. Introduction

Deep learning methods at present are playing an indispensable role in the field of computer vision. The relevant fruitful achievements also facilitate and change the fashion of synergy between clinical diagnosis and computerized assistance. The scope of application covers the demands of computer-guided pathological inspection [1,2,3,4], brain neural circuit mapping and tracking [1,5,6,7,8,9], specific tissue detection in image-based datasets [5,10,11,12,13], and other clinical applications. Among these applications, neural network (NN)-based recognition methods that are capable of detecting life-threatening abnormalities from image-based datasets especially attract the attention of both scientific and engineering participants [2,11,14,15,16,17] and gradually replace conventional approaches. Within these techniques, the emergence of fully convolutional neural networks (FCN) has successfully acquired more attention, and the FCN-based methods have further elevated the performance of convolutional neural networks (CNNs) in the field of modern medical image recognition and segmentation [15,18,19,20]. In applications of clinical practice, on the other hand, the medical image datasets are often sparse, so the technical development in these fields is leading by the NN models that are suitable for dealing with small-size datasets. Thus, starting from FCN, over V-net [15], U-net [21,22,23], and the auxiliary of feature extraction blocks [24,25,26], then to the attention U-net [27,28] and dimension fusion U-net (D-Unet) [29], the relevant methods gradually become the developing core in medical image analysis. Meanwhile, due to the extremely high mortality [1,13], the modality investigations of malignant brain tumors have also affected the mainstream techniques of the brain tumor image segmentation [1,30] and the procedures of image-guided surgery.

However, for pursuing the performance of model prediction, continuing to increase model depths and kernel numbers would become the burden of hardware architecture in the points of view of power control and the maintenance of computational complexity. For instance, the consumption of GPU memory and the computational complexity are all proportional to the size and number of convolutional kernels. Large numbers of parameters in training procedures also challenge the memory sizes of GPUs, the design of algorithmic schemes, and power requirements [14]. Additionally, the labor intervention and time cost in the stage of data labeling also obstruct the progression of technical development. Thus, the development of deep learning methods would step into the inevitable situations of high-cost hardware and high computational complexity configuration [14,16]. The combination of contour-based models and statistical-learning-based methods offer alleviant approaches by utilizing prior information [3,31,32,33,34] to reduce the high-cost scheme caused by the deep learning schemes. For instance, the statistical models of the random walks are used for tracking and segmenting thin and elongated image components by assigning seed points on the ends of these components [35]. Region-based contour models combined with statistical classification processes have been utilized to solve the problems of intensity inhomogeneity that occurred within image blocks [36,37,38] by arranging curve functions on the blocks of interest. Nevertheless, the manual intervention of the combinatorial approaches also causes different problems in artificially predefining labels of seeded image components [14,39,40,41]. Physical-based methods also offer another track to deal with these undergoing problems. The data density functional theory (DDFT) successfully extracts the intensity features from brain tumor image datasets by estimating the morphological heterogeneity in energy spaces [42,43] so that it may remove the undesired manual intervention. However, the architecture that can bridge the deep learning procedures and DDFT has not appeared yet.

Therefore, inspired by the merits of the physical framework of DDFT and also considering the advantages of deep learning methods, we introduced the Fermi–Dirac distribution to be a correction function in the following procedures. By employing the theoretical scheme of DDFT, we mapped the three-dimensional image space into a noninteracting physical system and treated each image voxel as a physical particle. Under this condition, the Fermi–Dirac distribution was used to analyze the particle behavior and classify these particle clusters according to their intrinsic energies. Thus, the particle clusters formed a specific morphological structure according to their energy property in the physical system. The estimates were then mapped back to the image space, and the image voxels were decomposed by considering their morphological heterogeneity. In the article, we compared the performance of the Fermi–Dirac correction function in Unet-based architectures. The experimental results validated the superiority of the proposed Fermi–Dirac correction function under the purpose of computational complexity reduction.

## 2. Datasets and Methods

### 2.1. Datasets

For fair comparisons, we employed the open datasets from the multimodal brain tumor segmentation challenge (BraTS) 2019 [44,45] to test the proposed algorithmic schemes. The type of datasets includes T1, T1-CE (T1-weighted contrast-enhanced), T2-weighted, and T2-FLAIR (Fluid-Attenuated Inversion Recovery). The datasets were skull-stripped, interpolated to the same resolution, and labeled by experienced neuro-radiologists. There are four labeled types for the tumor anatomic structures, which comprise the enhancing tumor (ET), the peritumoral edema, the necrotic and nonenhancing tumor core (TC), and the background. There are 300 testing sets and 35 validation sets, and 155 pieces of images in each individual set. The dimensions of each image size are 240 pixels both for height and width, and all of the images are in axial views.

### 2.2. Theoretical Scheme of Fermi–Dirac Correction Function

According to the theoretical framework of DDFT, an arbitrary high-dimensional image space can be isomorphically mapped into a pseudo-physical space with the same dimensionality once the data length is fixed [42]. Then, image voxel components are correspondingly mapped as particle-like clusters. Thus, there exists a bijective relation between the voxel intensity distribution and the Fermi hypersurface function. In other words, the voxel intensity can be mapped as a corresponding energy value within the pseudo-physical space. The voxel intensity distribution becomes a function of the Fermi hypersurface function under this theoretical framework, and vice versa. As the Fermi hypersurface function has an adequately theoretical definition in the pseudo-physical space [42], we could safely introduce appropriate physical properties to deal with the encountering situations. For instance, the Hamiltonian curves and the Lagrangian density functional could be exploited to measure the most-possible-cluster numbers and cluster boundaries, respectively [42,46]. Thus, we can delineate the morphological structure of images by applying these properties of energy under the framework of DDFT. For image segmentation, we introduced a specific particle distribution in terms of energy properties, named Fermi–Dirac distribution, to analyze and pre-decompose the image clusters according to their morphological structures.

Under the DDFT framework, the pseudo-physical space is spanned onto a noninteracting (or weakly interacting) system so that we can establish required conditions by considering each cluster to be a subsystem and each particle (mapped from a voxel in the image space) to be in a single-particle state. Additionally, as the locations and the corresponding intensity distribution of voxels within an image space are all fixed, there is no interaction between voxels, and this space can be treated as a stationary and “frozen” system. In other words, the clusters will not exchange particles and energy with their surroundings in the noninteracting system; thus, we can exploit an additional condition of zero temperature into this system. Thus, the probability of observing the number of particles n in the single-particle state can be estimated by introducing the perspective of thermodynamic properties:

(1) $$P_{n}=\frac{e^{-\left(n\varepsilon -n\varepsilon_{F}\right)/\varepsilon_{b}}}{Z},\;$$

where the parameters ε, $\varepsilon_{F}$, and $\varepsilon_{b}$ are the energy of the single-particle state, the Fermi energy at zero temperature, and the correction energy for dimensional balance, respectively. The symbol Z represents a partition function for the subsystem of interest:

(2) $$Z=\underset{n}{\sum }{\left[e^{-\left(\varepsilon -\varepsilon_{F}\right)/\varepsilon_{b}}\right]}^{n}.\;$$

The grand potential GV can be then defined by designating the particles to be pseudo-fermions or pseudo-bosons, respectively:

(3) $$GV=\left\{\begin{array}{c} \varepsilon_{b}\ln\overset{1}{\underset{n=0}{\sum }}Z=\varepsilon_{b}\ln\left\{1+e^{-\left(\varepsilon -\varepsilon_{F}\right)/\varepsilon_{b}}\right\},\;\mathrm{for}\;\mathrm{pseudo}-\mathrm{fermions}\\ \varepsilon_{b}\ln\overset{\infty }{\underset{n=0}{\sum }}Z=-\varepsilon_{b}\ln\left\{1-e^{-\left(\varepsilon -\varepsilon_{F}\right)/\varepsilon_{b}}\right\},\;\mathrm{for}\;\mathrm{pseudo}-\mathrm{bosons}\; \end{array}\right..$$

Then, the corresponding particle distribution functions for each particle type can be expressed formally:

(4) $$\hat{n}=\frac{\partial GV}{\partial \varepsilon_{F}}=\left\{\begin{array}{c} \frac{1}{e^{\left(\varepsilon -\varepsilon_{F}\right)/\varepsilon_{b}}+1},\;\mathrm{for}\;\mathrm{pseudo}-\mathrm{fermions}\\ \frac{1}{e^{\left(\varepsilon -\varepsilon_{F}\right)/\varepsilon_{b}}-1},\;\mathrm{for}\;\mathrm{pseudo}-\mathrm{bosons}\; \end{array}.\right.\;$$

The expressions of these distributions are the well-known Fermi–Dirac (FD) distribution and Bose–Einstein (BE) distribution, respectively. Equation (4) lists their representations in our proposed pseudo-physical system. It should be emphasized that the n takes 0 and 1 for pseudo-fermions and 0, 1, 2, …, ∞ for pseudo-bosons. Theoretically, the maximum of n in different particle types (fermions or bosons) represents the maximum capacity of particles a single-particle state can contain.

**Figure 1:**
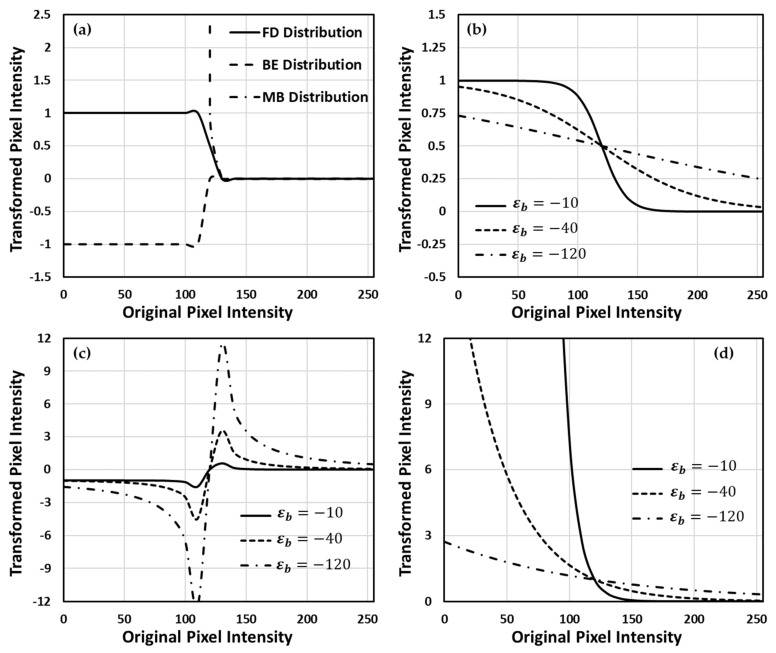
The model estimations of proposed distributions. In the model estimations, the global mean value is $\varepsilon_{F}=125$ and the value $\varepsilon \in \left[1,255\right]$. (**a**) Difference comparison of the three proposed distributions, and the minus global standard deviation is $\varepsilon_{b}=-1$. (**b**) Behavior of the Fermi–Dirac (FD) distribution with different $\varepsilon_{b}$ values. (**c**,**d**) The same situations but with Bose–Einstein (BE) and Maxwell–Boltzmann (MB) distribution, respectively.

We found an interesting phenomenon by simultaneously comparing the theoretical form of the FD distribution to that of the sigmoid function and the z-score normalization. The coarse form of the FD distribution is exactly the sigmoid function, so the pseudo-particle distribution will be squeezed into the range of $\left[0,1\right]$ as expected. On the other hand, as the form of the mathematical kernel of the FD distribution,$\;\left(\varepsilon -\varepsilon_{F}\right)/\varepsilon_{b}$, is also similar to the z-score normalization, we found a route to determine well the parameter definitions of pseudo-energy by comparing their parameters. Due to the voxel intensity in the image space and the energy in the pseudo-physical space being bijective, we correspondingly assigned these parameters, ε, $\varepsilon_{F}$, and $\varepsilon_{b}$, to be the intensity value distribution of each image, the global mean intensity of each testing set, and the minus global standard deviation of intensity of each testing set, respectively. Thus, the FD distribution exhibits its own mathematical merits for data processing by fusing the characteristics of the sigmoid function and the z-score normalization. Mathematically, we can clarify the difference between the FD and the BE distributions by examining their behavior under extreme conditions. When the value of $\varepsilon -\varepsilon_{F}$ in the kernel approaches zero, it also means that the intensity approaches its mean value in the subsystem, the value of the FD distribution will reach 0.5, whereas that of the BE distribution will approach infinity. Thus, the BE distribution intrinsically reveals its own anti-behavior compared to the sigmoid function. When the kernel is much larger than 1, both FD and BE distributions will have the same mathematical form $\hat{n}\approx e^{-\left(\varepsilon -\varepsilon_{F}\right)/\varepsilon_{b}}$. This form is also a well-known Maxwell–Boltzmann (MB) distribution. Figure 1 illustrates each mathematical behavior of the mentioned distributions with different values of minus global standard deviation $\varepsilon_{b}$.

As the bosons are allowed to have the same energy value, it would lead to a consequence that all of the voxels are classified in the same cluster and become indistinguishable. On the other hand, the intrinsic mathematical form of the BE distribution also causes itself to be an infinite value while the voxel intensity approaches its mean value; thus, these inconvenient properties make the BE distribution hardly a correction function for data normalization and data filtering in the data processing. A similar situation would happen to the MB distribution. Additionally, it is also impracticable to estimate the mean intensity of each subsystem technically due to the undesired computational complexity; thus, we utilized the global mean intensity of the whole system to conquer this issue. Therefore, by considering both the theoretical and the technical merits, the FD distribution is more suitable as a correction function in the data preprocessing procedures.

### 2.3. Experimental Framework

To test the performance of the proposed FD correction function, we employed the conventional preprocessing methods, a z-score normalization function, a Gamma correction function, and a three-dimensional (3D) global histogram equalization function [47], for the performance comparison and used a null-preprocess as a baseline. As mentioned above, the mathematical structure of the FD correction function was established by a coarse skeleton and a kernel embedded in the skeleton. Theoretically, the skeleton is a sigmoid function, and the kernel function has the same form as the z-score normalization function. Thus, we adopted the z-score normalization function as one of the preprocessing ways for a fair comparison of performance and then investigated the differences between these functions. On the other hand, we also adopted the Gamma correction function due to its performance being adjustable by assigning an appropriate parameter. Thus, we can also compare the performance of conventional supervised correction functions to the proposed FD correction function. The way to determine the optimized Gamma parameter directly relies on a large number of experiments. We extracted the Gamma parameters in a set of $\left\{0.2,\;0.4,\;0.6,\;0.8,\;1.0,\;1.2,\;1.5,\;2\right\}$. According to the performance of image segmentation, we adopted the value of 0.6 as the Gamma parameter due to it offering the best performance in the image segmentation. Finally, we compared the capability between the global histogram equalization function and our functions on the topic of image augmentation.

**Figure 2:**
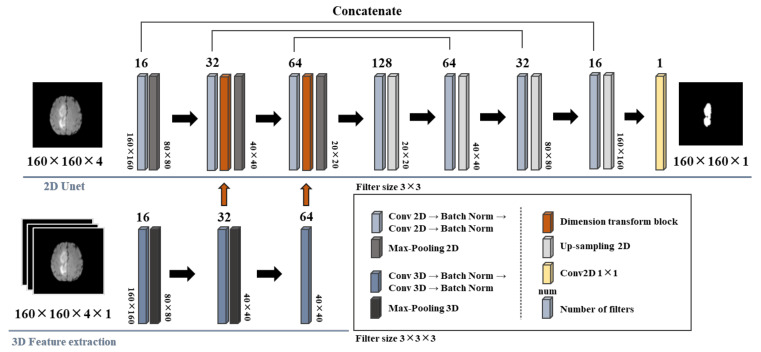
The framework of the D-Unet-based structure for the brain tumor image segmentation. The original image sizes are 240 pixels, and we rescaled the input sizes to 160 pixels to match the requirements of the adopted neural network models. The third-dimensional value of 160 × 160 × 4 used in the 2D Unet represents four-type images, include T1, T1-weighted contrast-enhanced (T1-CE), T2-weighted, and T2-fluid-attenuated inversion recovery (FLAIR), and so does that used in the 3D feature extraction procedure. The fourth dimension value of 160 × 160 × 4 × 1 in the 3D feature extraction procedure represents the number of trials. The dimension-transform-blocks were used to fuse the two- and three-dimensional features in the encoding procedure, and only fused- and two-dimensional features were used for the information decoding. Thus, these procedures can offer a trade-off between high-dimensional information and computational complexity.

Thus, five preprocessing scenarios were employed for the performance comparison under a specific neural network model. To achieve this purpose, we utilized the D-Unet-based structure for the brain tumor image segmentation. Figure 2 illustrates the relevant framework. D-Unet is established based on the conventional U-net structure, and thus, its structure is suitable for dealing with small-size datasets. Furthermore, a delicate function component named the dimension-transform-block [29] is employed in the encoding procedure of D-Unet, then all of the two- and three-dimensional extracted features would be fused through these blocks. As illustrated in Figure 2, two dimension-transform-blocks (indicated as the red columns) were used in the proposed D-Unet scheme. Meanwhile, to reduce the undesired computational cost, only the fused features and the two-dimensional features are fed into the decoding procedure. Thus, the D-Unet structure can simultaneously preserve the significant high-dimensional features and offer an acceptable computational cost. Under the framework of the employed D-Unet structure, the dimensions of image sizes were scaled down to 160 pixels both for height and width, and the input types of each image included four channels, T1, T1-CE, T2-weighted, and T2-FLAIR. The adopted numbers of filters are indicated above each layer, and only one channel, the whole tumor (WT), would be output for the performance comparison. The output activation layer was a sigmoid function. The number of epochs and batch size were set to 30 and 32, respectively, for all preprocessing scenarios. The optimizer and the loss function were Adam and the three-dimensional soft dice loss function [48,49], respectively. The analytic form of the soft dice loss function is [15,48,49,50]:

(5) $$DL=1-\frac{2\sum_{i}^{N}p_{i}g_{i}+\epsilon }{\sum_{i}^{N}p_{i}^{2}+\sum_{i}^{N}g_{i}^{2}+\epsilon }.$$

The number N is the total data length and the symbol $\epsilon =10^{-5}$ was adopted to avoid DL diverging. The parameters $p_{i}\in \left\{0,1\right\}$ and $g_{i}\in \left\{0,1\right\}$ are respectively the binary predicted segmentation and ground truth labeling. Ref. [50] offers the source codes of the soft dice loss. The initial value of the learning rate of all scenarios is set to 0.00015. The relevant model parameters and functions are collected and listed in Table 1.

## 3. Results

**Figure 3:**
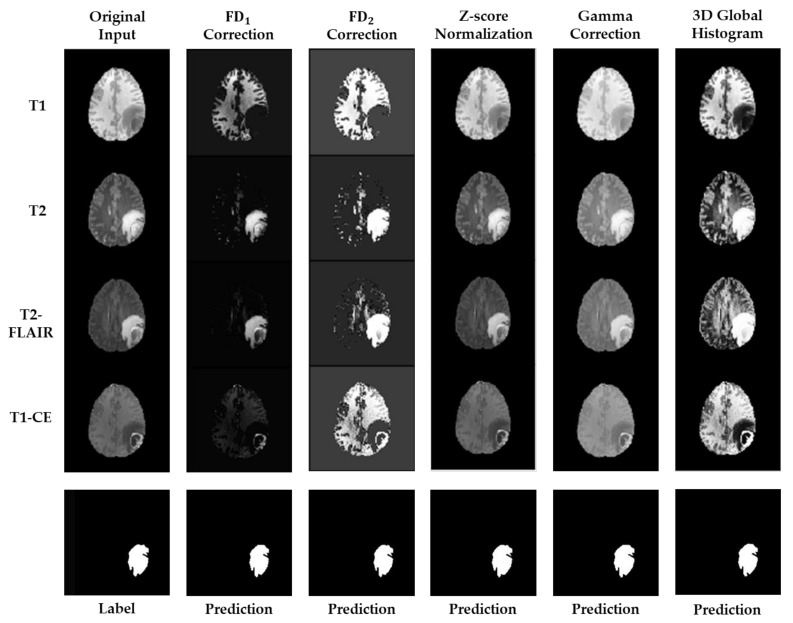
The figure shows the visualized results from the employed preprocessing methods and the proposed FD-type correction functions. Two values of $\varepsilon_{b}$ were individually employed to estimate the kernel, and the corresponding results are illustrated in the second and third columns. The proposed FD-type correction functions exhibit the capability not only for image intensity normalization but also for image component filtering. The following columns list the preprocessed results using the other employed methods.

All of the experiments were executed with the hardware specification of i9-9980XE CPU @ 3 GHz, 18 cores, and one GPU of NVIDIA GeForce RTX 2080 Ti. Figure 3 illustrates the visualized comparison between the employed preprocessing methods and the proposed FD correction function. To theoretically inspect the capability of the FD correction function, two values of $\varepsilon_{b}$ were used to construct the kernel. The corresponding values for ${\mathrm{FD}}_{1}$ and ${\mathrm{FD}}_{2}$ correction functions were $\varepsilon_{b}=-1$ and $\varepsilon_{b}=$ minus the global standard deviation of intensity of each testing set, respectively. In the words, the ${\mathrm{FD}}_{2}$ correction function is a standard FD correction function. To clarify the main difference between ${\mathrm{FD}}_{1}$ and ${\mathrm{FD}}_{2}$ correction functions, their analytical forms are:

(6) $${\mathrm{FD}}_{1}=\frac{1}{e^{-\left(\varepsilon -\varepsilon_{F}\right)}+1}{\;\mathrm{and}\;\mathrm{FD}}_{2}=\frac{1}{e^{-\left(\varepsilon -\varepsilon_{F}\right)/|\varepsilon_{b}|}+1}.$$

By comparing to the results of other preprocessing methods, all of the proposed FD-type correction functions can remove the insignificant components of the brain tissue images, especially for the cases of T2 and T2-FLAIR images. Meanwhile, the ${\mathrm{FD}}_{2}$ correction function can further enhance the filtered image components, and so did the 3D global histogram equalization function. The z-score normalization and the Gamma correction functions exhibit similar preprocessing results.

The performance comparison of all preprocessing scenarios is listed in Table 2. The dice score of WT without any preprocessing is the lowest one as expected, and thus, we used its corresponding computational time as the baseline. All dice scores of the employed preprocessing methods are similar, and the FD-type correction functions present slightly high scores. Even though the dice score of the z-score normalization function reaches a good level, its computational time is much higher than that of baseline. It might be that the execution efficiency of this method was not optimized. On the other hand, the Gamma correction function also exhibits good performance in both the dice score and the corresponding computational time. However, it costed a large number of experiments and labor intervention to choose the optimized gamma parameter. In our experiments, the final optimized parameter of the Gamma correction function was 0.6. Although the dice score of the 3D global histogram equalization function is the lowest one compared to all employed methods, its computational time reveals the superior performance. Table 3 represents a confusion-matrix table to show the comparison of accuracy, sensitivity (recall), and precision between the proposed FD-type correction functions and the conventional correction methods. The definition of each indicator is as follows [51,52]:

(7) $$\mathrm{Accuracy}=\frac{\mathrm{TP}+\mathrm{TN}}{\mathrm{TP}+\mathrm{TN}+\mathrm{FP}+\mathrm{FN}},$$

(8) $$\mathrm{Recall}\left(\mathrm{Sensitivity}\right)=\frac{\mathrm{TP}}{\mathrm{TP}+\mathrm{FN}},\;$$

(9) $$\mathrm{Precision}=\frac{\mathrm{TP}}{\mathrm{TP}+\mathrm{FP}}.\;$$

The factors TP, TN, FP, and FN are the true positive, true negative, false positive, and false negative, respectively. The total summation of these four factors is around 812,535 with a small residual error. Then, the total number of tumors is defined as TP+FN and has a value of about 21,191.

**Figure 4:**
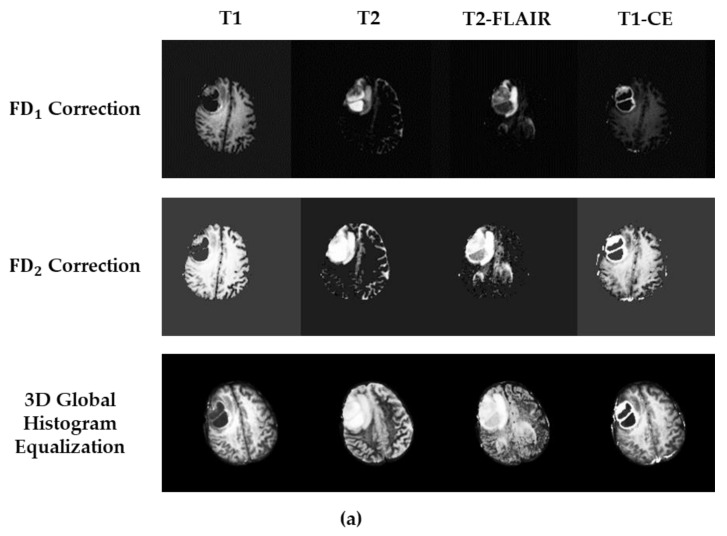

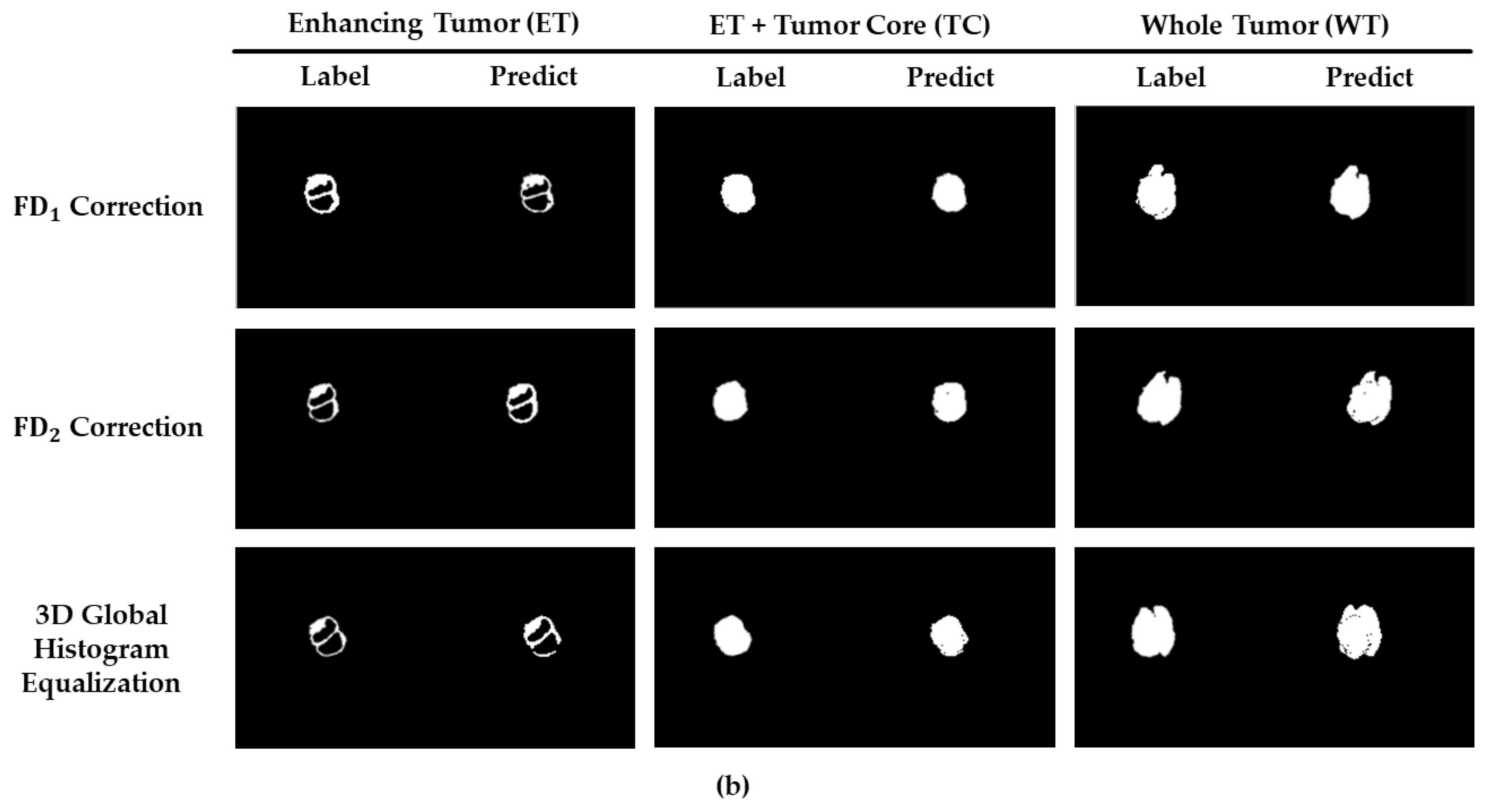
The visualized comparison between the 3D global histogram equalization function and the proposed FD-type correction functions. (**a**) The preprocessing results dealing with these functions, and (**b**) the predicted results of the tumor image segmentation categorized into ET, TC, and WT, and their corresponding ground truths.

Thus, we further compared the performance between the 3D global histogram equalization function and the proposed FD-type correction functions, wherein the tumor labels were categorized into WT, TC, and ET in detail. The output channels of the D-Unet became three branches. Table 4 and Table 5 respectively list the performance comparison and the corresponding confusion-matrix table. We preserved all of the model parameters for a fair comparison but replaced the output layer with a softmax activation function for multi-label detections. We also presented the corresponding dice scores of each label for the training stage and the validation stage. As expected, the dice scores of the validation stage are much lower than those of the training stage. All computational times in this experiment are comparable, but the required time costs were all raised due to the multi-label detections. In this experiment, all of the dice scores of the FD-type correction functions are higher than that of the 3D global histogram equalization function. We might deduce these reasons by utilizing their visualized results, as illustrated in Figure 4.

All types of image sets were processed with the 3D global histogram equalization function and the proposed FD-type correction functions, and the preprocessed images are illustrated in Figure 4a. The image inclinations resulted from the augmentation of affine transformations. Then, the corresponding results of brain tumor image segmentation are illustrated in Figure 4b. As the tumor recognition of ET type is typically hard, the corresponding dice scores are quite low, as listed in Table 3, for all employed preprocessing methods. The visualized results of the ET type illustrated in Figure 4b reflect this fact. We can recognize that the image structures of ET type are meticulous compared to TC and WT, and thus, that it would cause difficulty in the ET tumor image recognition. It should also be emphasized that the segmented results of TC and WT types are similar in all employed methods in the experiments. Then, the ${\mathrm{FD}}_{2}$ correction function shows better performance in recognizing the ET tumor edges than the other two methods. Therefore, the results validate that the ${\mathrm{FD}}_{2}$ correction function, i.e., the FD correction function with a kernel of z-score normalization function, exhibits superior performance in both computational time reduction and brain tumor image segmentation by comparing other conventional preprocessing methods.

## 4. Discussion and Conclusions

We theoretically reconstructed the Fermi–Dirac distribution function to be a correction function for brain tumor image segmentation. The skeleton form and the kernel of the proposed Fermi–Dirac correction function are respectively a sigmoid function and a z-score normalization function. Thus, the Fermi–Dirac correction function inherits the merits of these functions. It is suitable for dealing with intensity normalization and filtering insignificant image components simultaneously. We also explained the mathematical inconvenience of the Bose–Einstein and Maxwell–Boltzmann distribution functions in imaging processing.

We then compared the performance of the proposed correction function with other conventional preprocessing methods, such as the standard z-score normalization, the Gamma correction, and the three-dimensional global histogram equalization. We also changed the normalization factor of the kernel to inspect the mathematical properties of the proposed Fermi–Dirac correction functions. The experimental consequences validated that the proposed correction function can have better computational cost than those of conventional and supervised methods. Then, the proposed correction function would inhibit the insignificant image components and enhance the filtered image segments. As the experimental results also showed that the three-dimensional global histogram equalization can offer a similar computational capability as the proposed correction method, we further fed those methods into a complete comparison. The experimental results validate the superiority of the proposed Fermi–Dirac correction function.

In further performance comparison, the proposed Fermi–Dirac correction function exhibits its superior computational and recognizing capability for the brain tumor image segmentation with the multimodal types of ET, TC, and WT. Therefore, the proposed Fermi–Dirac correction function can not only reduce the computational complexity but also reinforce the image component recognition and segmentation.

## Figures and Tables

**Table 1:** Model parameters and relevant functions.

Parameter/Function	Value/Method
Number of epochs	30
Batch size	32
Learning rate (Initial value)	0.00015
Loss function	3D soft dice loss function
Optimizer	Adam

**Table 2:** Performance comparison between the conventional preprocessing methods and the proposed FD-type correction functions. This table only lists the dice scores of the whole tumor (WT). Numbers in bold show the best records.

Preprocessing Method	Dice Score (WT only)	Computational Time (min)
Null	0.7183	91
z-score Normalization	0.9296	142
3D Global Histogram Equalization	0.9148	**84**
Gamma Correction ^1^	0.9319	93
${\mathrm{FD}}_{1}$ correction	**0.9431**	88
${\mathrm{FD}}_{2}$ correction	0.9347	90

^1^ The gamma parameter = 0.6.

**Table 3:** The confusion-matrix table. It shows the comparison of accuracy, sensitivity (recall), and precision between the proposed FD-type correction functions and the conventional correction methods. Numbers in bold show the best records.

Preprocessing Method	Validation Stage (WT Only)						
	TP	TN	FP	FN	Accuracy	Recall	Precision
Null	15,306	789,342	2003	5886	0.9901	0.71	0.85
z-score Normalization	19,512	789,570	1776	1680	0.9957	**0.90**	0.90
3D Global Histogram Equalization	19,293	789,400	1947	1899	0.9952	0.89	0.89
Gamma Correction ^1^	18,603	790,100	1248	2562	0.9953	0.86	**0.92**
${\mathrm{FD}}_{1}$ correction	19,180	790,010	1339	2012	**0.9959**	0.89	0.91
${\mathrm{FD}}_{2}$ correction	19,471	789,500	1841	1721	0.9956	**0.90**	0.89

^1^ The gamma parameter = 0.6.

**Table 4:** Further performance comparison between the 3D global histogram equalization function and the proposed FD-type correction functions. The dice scores of the tumor components categorized as WT, tumor core (TC), and enhancing tumor (ET) are individually presented for the comparison. Numbers in bold show the best records.

Preprocessing Method	Dice Score			Computational Time (min)
		Training Stage	Validation Stage	
3D Global Histogram Equalization	WT:	0.9220	0.8002	138
	TC:	0.9419	0.7688	
	ET:	0.9142	0.6365	
${\mathrm{FD}}_{1}$ correction	WT:	0.9491	0.8337	140
	TC:	0.9757	0.7976	
	ET:	0.9559	0.6802	
${\mathrm{FD}}_{2}$ correction	WT:	0.9336	**0.8433**	141
	TC:	0.9773	**0.8041**	
	ET:	0.9606	**0.6848**	

**Table 5:** The confusion-matrix table. Numbers in bold show the best records.

Preprocessing Method		Validation Stage						
		TP	TN	FP	FN	Accuracy	Recall	Precision
3D Global Histogram Equalization	WT:	18,910	789,880	1465	2281	0.9953	0.89	0.89
	TC:	7818	801,309	1490	1918	0.9958	**0.80**	0.84
	ET:	3141	807,914	700	780	0.9981	0.80	0.82
${\mathrm{FD}}_{1}$ correction	WT:	18,949	789,874	1470	2242	0.9954	0.89	**0.93**
	TC:	7627	801,691	1107	2110	0.9960	0.78	0.87
	ET:	3007	808,156	459	914	0.9983	0.77	0.87
${\mathrm{FD}}_{2}$ correction	WT:	19,240	789,955	1340	1952	**0.9959**	**0.91**	**0.93**
	TC:	7966	801,841	958	1772	**0.9966**	**0.82**	**0.89**
	ET:	3153	808,200	415	768	**0.9998**	**0.80**	**0.88**

## Data Availability

Publicly available datasets were analyzed in this study. This data can be found here: https://www.med.upenn.edu/cbica/brats2019/data.html.
